# Modeling the determinants of smoking behavior among young adults in Khuzestan province: a two-level count regression approach

**DOI:** 10.3389/fpubh.2024.1449193

**Published:** 2024-11-28

**Authors:** Homayoun Satyar, Kambiz Ahmadi Angali, Somayeh Ghorbani, Naser Kamyari, Maryam Seyedtabib

**Affiliations:** ^1^Department of Biostatistics and Epidemiology, School of Health, Ahvaz Jundishapur University of Medical Sciences, Ahvaz, Iran; ^2^Department of Biostatistics and Epidemiology, School of Health, Social Determinants of Health Research Center, Ahvaz Jundishapur University of Medical Sciences, Ahvaz, Iran; ^3^Cancer Research Center, Golestan University of Medical Sciences, Gorgan, Iran; ^4^Department of Biostatistics and Epidemiology, School of Health, Research Center for Environmental Contaminants (RCEC), Abadan University of Medical Sciences, Abadan, Iran

**Keywords:** smoking behavior, young adults, Khuzestan, count regression models, zero-inflated models

## Abstract

**Purpose:**

This study investigates the determinants of smoking behavior among young adults in Khuzestan province, southwest Iran, using two-level count regression models. Given the high prevalence of smoking-related diseases and the social impact of smoking, understanding the factors influencing smoking habits is crucial for effective public health interventions.

**Methods:**

We conducted a cross-sectional analysis of 1,973 individuals aged 18–35 years, using data from the Daily Smoking Consumption Survey (DSCS) in Khuzestan province collected in 2023. A variety of count regression models, including Poisson, Negative Binomial, Conway–Maxwell Poisson, and their zero-inflated counterparts, were evaluated. The best-fitting model was selected based on goodness-of-fit indices.

**Results:**

Approximately 90% of participants were non-smokers. Among smokers, the prevalence of light, moderate, and heavy smoking was 47.7, 19.0, and 33.3%, respectively. The two-level Zero-Inflated Conway–Maxwell Poisson (ZICMP) model provided the appropriate fit for the data. Key determinants of daily cigarette consumption included gender, age, education, and Body Mass Index (BMI). Men consumed 3.24 times more cigarettes per day than women. Higher education levels were inversely related to smoking intensity, with MSc/PhD holders having significantly lower smoking rates. Age and BMI also significantly influenced smoking behavior, with younger and obese individuals showing lower smoking rates.

**Conclusion:**

The use of advanced count models capable of handling numerous zeros and overdispersion is crucial for accurately analyzing trends in cigarette consumption across different population groups. The results indicate that factors such as older age, lower education levels, and gender differences influence smoking behavior. Therefore, prevention strategies aimed at delaying the onset of smoking, particularly among men, and promoting education among adolescents can effectively reduce smoking rates. However, further research should consider additional socioeconomic variables and encompass a broader age range to enhance the understanding of smoking behavior.

## 1 Introduction

Smoking is a leading cause of preventable deaths globally and is one of the main risk factors for developing various diseases, particularly non-communicable diseases such as cardiovascular and respiratory diseases, cancer, and stroke (1). Smoking significantly diminishes health-related quality of life and causes over 5 million deaths worldwide each year (2). The general lifestyle of a heavy smoker is poorer than that of a non-smoker (3). Smoking has a significant impact on healthcare systems and national economies due to increased medical service usage, higher absenteeism from work, and greater costs (2). Additionally, tobacco use is a leading cause of early death, responsible for approximately 9% of global deaths (4). According to the Global Burden of Disease Study in 2015 smoking was the second leading cause of death and disability in central Europe (5) and considered it the most preventable cause of death and disease (6–8). Globally in 2019, around 8.7 million deaths were attributed to tobacco use, including smoked, second-hand, and chewing forms (9). Long-term tobacco use contributes to risky behaviors, psychological problems, and physical health decline (10). Previous studies have shown that smoking just a few cigarettes during adolescence increases the risk of smoking by 16 times in adulthood (11). Moreover, starting to smoke at a younger age is associated with greater difficulties in quitting smoking as an adult (12). Even smoking just one cigarette a day can increase a person’s heart rate and blood pressure (13).

Global studies indicate a rising prevalence of smoking among young people, particularly in developing countries, with the age of initiation decreasing (14). Furthermore, as the age of onset of smoking decreases, the frequency of smoking in adulthood increases (15). Having a smoker in the family significantly increases the risk of teenagers smoking (16). According to the World Health Organization in 2011, 6 million deaths annually are attributed to tobacco use, with 1.5 million of these deaths occurring in women. It is predicted that by 2030, deaths due to smoking will reach 8 million per year (17, 18).

According to the WHO report for 2020, there are 1.3 billion tobacco users worldwide, including 7.8% of women and 39.7% of men (19). Some demographic and individual factors such as age, gender, education, occupation and etc.; have an influence on smoking behavior (20). The studies showed that cigarette consumption is different in age grouped. In other word, smoking intensity increases with age (21). According to studies, the prevalence of smoking is significantly lower in women than in men (22). However, it is reported that the decline in smoking is slower in women than in men (23). Education also has a significant influence on smoking rates, as studies show that a higher level of education correlates with a lower prevalence of smoking (24). In addition, many studies have investigated the relationship between various diseases and smoking. According to a study, people with mental health conditions are twice as likely to smoke as those without such conditions, indicating a strong link between the desire to smoke and the intensity of smoking with mental health (25, 26). Also, research has investigated the link between smoking and chronic diseases such as diabetes and found that smoking contributes to increased mortality in diabetics (27). On the other hand, a history of cardiovascular disease in the family is a protective factor for smoking. Smokers report consuming 15% fewer cigarettes than those who have no such history in the family (20). Obesity has been investigated as another variable related to smoking in studies. A study has shown that people who smoke are less obese than people who do not smoke at all (28).

Several studies indicate the prevalence of smoking among the population in Iran (29–31). According to the 2010 estimate by the World Health Organization, approximately 12% of Iran’s adult population were daily smokers, and this figure was reported as 10% in 2015 (32, 33). A study in 2020 showed that the overall prevalence of smoking in Iran is 12%, with rates of 23.4% in men and 1.4% in women (10), and it has been rising in recent decades.

Khuzestan province in southwestern Iran is the fifth most populous province, with 29 cities and around 4,700,000 residents as of the 2015 census. It has a diverse population, including Fars, Arab, Lur, Bakhtiyari, Qashqai Turk, Kurd. According to the National STEPs Survey 2016, the prevalence of smoking in the province was 9.1%, with 0.3% among women and 17.3% among men (10). Given the health, social, and economic consequences of smoking, along with the province’s strategic location, it is essential to comprehend the smoking behaviors and patterns of young adults in this region. Additionally, the cultural context of Khuzestan may impact these patterns.

Smoking intensity, often measured by the number of cigarettes smoked per day, is considered a count response. Therefore, count regression models are appropriate for analyzing the factors that influence smoking intensity (6, 34). No single model is perfect for this type of data. Thus, we examine six count models in this study, including Poisson (P), Negative Binomial (NB), Conway–Maxwell Poisson (CMP), and the corresponding zero-inflated (ZI) models (ZIP, ZINB, and ZICMP). The ZINB and ZICMP models can simultaneously handle overdispersion and excess zeros in count regression data that may be present in this study (35). Due to possible differences in the prevalence of smoking in cities within the province, it is also necessary to consider the dispersion and heterogeneity of smoking consumption. Therefore, two-level (TL) count models with many zeros are used in this study to analyze the factors related to smoking in Khuzestan province and compare these models using goodness-of-fit indices.

## 2 Materials and methods

### 2.1 Data

This cross-sectional study utilized data from the 2023 Daily Smoking Consumption Survey (DSCS) in Khuzestan province (36), targeting individuals aged 18 to 35 years. A multi-stage cluster sampling method was used for the survey. In the first stage, the cities were selected as clusters. Within each city, public and highly frequented places such as parks, shopping centers and main streets were then selected as sub-clusters. The people who spent time in these places were selected as sampling units. Finally, the desired sample was randomly selected from the people in these locations in proportion to the population of the respective city. Of the nearly 5,100 samples in the survey, we only included people between the ages of 18 and 35. After cleaning the data and removing questionnaires with incomplete responses, 1,973 out of 2,112 were finally considered for the current study.

A paper questionnaire was used to collect the required information. The questionnaire collected information on demographic status, personal details, and behavioral risk factors. The response variable was the number of cigarettes consumed daily.

Independent variables included area of residence (rural/urban), age (18–35 years), sex (male/female), Body Mass Index (Underweight: <18.5, Normal: 18.5–24.9, Overweight: 25–29.9, Obese: 30–34.9, Extremely obese: ≥35 kg/m^2^), education (elementary/middle, diploma, AD/BSc, MSc/PhD), marital status (married/single), occupation (unemployed or student/organizational/freelance/agricultural or livestock/housewife), general study (yes/no), hookah consumption (yes/no), family chronic disease history (yes/no), family mental disease history (yes/no), and disease history (yes/no). Chronic diseases included blood pressure, diabetes, heart disease, kidney disease, and respiratory disease. General study refers to reading and learning from non-academic texts like articles, journals, novels, websites, and other informal writings. Smoking severity is categorized into three levels: light (1–5 cigarettes per day), moderate (6–10 cigarettes per day) and heavy smoking (>10 cigarettes per day). Cases where individuals had smoked in the past but were non-smokers at the time of this study were considered non-smokers.

### 2.2 Statistical model

We employed Poisson, Negative Binomial, and Conway–Maxwell–Poisson models, as well as their zero-inflated versions, to analyze the data. Each distribution is described in detail below.

#### 2.2.1 Poisson model

The Poisson probability distribution of the response variable (*y*) is as follows:


$$f\left(y;\mu \right)=\frac{\exp\left(-\mu \right)\mu^{y}}{y!},\;y=0,1,2,3,\ldots$$

that *μ* > 0, representing the mean and variance of the response variable. To measure the effect of explanatory variables on the response, the natural logarithm of the mean parameter *μ* is modeled based on the given pattern of explanatory variables (37).

#### 2.2.2 Negative binomial model

The probability distribution of the Negative Binomial is defined as:


$$\begin{array}{l} f\left(y;\;r,\;p\right)=\left(\begin{array}{c} r+y-1\\ y \end{array}\right)p^{r}{\left(1-p\right)}^{y};\;\;\\ y=0,1,2,\ldots \;r>0\;\mathrm{and}\;0<p<1 \end{array}$$

where, $E\left(y\right)=\frac{r\left(1-p\right)}{p}$ and $V\left(y\right)=\frac{r\left(1-p\right)}{p^{2}}$ indicate the mean and variance of the response variable. The canonical link function used to model response variable and covariates is log ( $\frac{1-p}{p}$ ). The parameter r is known as dispersion (over-dispersion) parameter (38).

#### 2.2.3 Conway–Maxwell Poisson

The probability mass function of the Conway–Maxwell Poisson (CMP) distribution is given by:


$$p\left(Y=y\;|,\;\lambda |,\nu \;\right)=\frac{\lambda^{y}}{{\left(y!\right)}^{\nu }}\times \frac{1}{Z\left(\lambda ,\nu \right)},\;y=0,1,2,\ldots \;\;\lambda >0$$

where, $Z\left(\lambda ,\nu \right)=\overset{\infty }{\underset{i=0}{\sum }}\frac{\lambda^{i}}{i{!}^{\nu }}$ is normalizing constant in the regular CMP distribution. The dispersion parameter underlying this distribution is denoted by ν , where ν≥0 . A value of [0,1) indicates over-dispersion in the data, and for *ν* > 1 under-dispersion occurs. The CMP distribution reduces to the Poisson distribution, when ν=1 (39).

### 2.3 Zero-inflated models

In some cases, datasets contain an excessive number of zeros that cannot be adequately described using conventional count distributions. To address this issue, modifications to the count models are made to account for the excess zeros. Zero-inflated count models are two-part models, consisting of binary and count model components. Both the binary and count components account for the zero counts, while the count component addresses the count outcomes.

It is assumed that the data arise from a mixture of two distributions: structural zeros generated by a binary distribution and non-negative integer outcomes (including zeros) produced by a count distribution. In zero-inflated models, the binary component is usually represented by a logit model, using logistic regression to model the structural zeros. Poisson or negative binomial regression is usually used for the count results. The general structure of a Zero-Inflated distribution is as follows:


$$P\left(Y=y\right)=p+\left(1-p\right)f\left(y|\theta \right)\;y=0,1,2,3,\ldots$$

where p is the probability of an excess zero and $f\left(y|\theta \right)$ is the probability mass function of a common count distribution (35).

### 2.4 Multilevel zero-inflated count regression model

We consider a count distribution for the response variable ( $y_{ij}$ ), where ( $i=1,2,\ldots ,m;j=1,2,\ldots ,n_{i}$ ) and $y_{ij}$ indicating the count of cigarettes smoked by the *j*th person in the *i*th city. The multilevel (two-level) regression model is as follow:


$$\log\left[\frac{\phi_{ij}}{1-\phi_{ij}}\right]=\xi_{ij}=a_{ij}^{T}\alpha +w_{i}$$

$$\log\left[\lambda_{ij}\right]=\eta_{ij}=b_{ij}^{T}\beta +u_{i}$$

where $a_{ij}^{T}$ and $b_{ij}^{T}$ are the fixed effect covariates related to *j*th observation in the *i*th cluster (city), and $w_{i}$ and $u_{i}$ are the city-specific random effects in the zero and count parts of the model, respectively. The random effects *w* and *u* are assumed to be independent and normally distributed with mean zero and variance $\sigma_{w}^{2}$ and $\sigma_{u}^{2}$ , respectively (40, 41).

In the two-level regression model, individuals are in the first-level that nested within the city, which are the second-level. Twenty-nine cities of Khuzestan province are considered at the second level in this study with cluster sizes varying from 5 to 235. Six models, including Poisson (P), Negative Binomial (NB), Conway–Maxwell Poisson (CMP), Zero-inflated Poisson (ZIP), Zero-inflated Negative Binomial (ZINB), and Zero-inflated Conway–Maxwell Poison (ZICMP) were considered for data analysis.

The results of the models were reported in terms of *β*, odd ratio (OR) and, rate ratio (RR). OR indicates the chance of becoming a smoker in each group compared to the reference group. The RR is the ratio of the probability of an outcome (daily cigarettes smoked) in an exposed group to its probability in an unexposed group (reference group), calculated as $\exp\left(\beta \right)$ . All models are compared using several main indices including Log-Likelihood (LL), Deviance (D), Akaike Information Criterion (AIC), Bayesian Information Criterion (BIC), and Mean Square Error (MSE). All analysis was performed with R software 4.2.2 with package GlmmTMB (42) and GraphPad Prism 8.

## 3 Results

**Figure 1:**
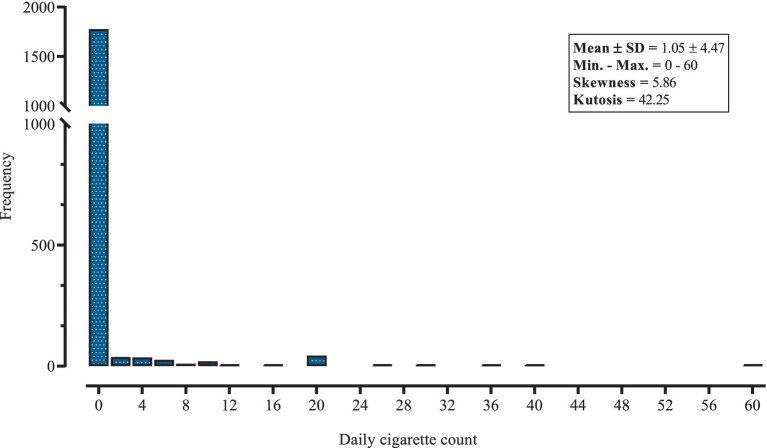
Frequency distribution of daily cigarette consumption among the 18–35 age group.

The frequency distribution of the data provides a comprehensive overview of the daily cigarette consumption of respondents in Khuzestan province (Figure 1). The mean (SD) of cigarettes smoked daily by Khuzestan residents was 1.05 (4.47), which shows that the variance is almost 19 times higher than the mean. In the smoking population, the frequency of heavy smokers has decreased. This distribution illustrates the skewed nature of smoking habits within the population, with a considerable majority either not smoking at all or smoking very few cigarettes per day. In the city of Dehdez, the average daily cigarette consumption was 3.79 (9.23), while in Lali it was 2.50 (7.07), the highest in the province. In addition, the prevalence of smoking was highest in Dehdez at 21.4%, while it was zero in the two cities of Andika and Hendijan (Table 1). Also, the prevalence of light, moderate and heavy smoking were 4.71, 1.88 and 3.29%, respectively. The descriptive statistics and comparisons of response components (zero value or count value) by variable are shown in Table 2, such that significant differences in smoking behavior were observed across various demographic and lifestyle factors. The results showed that the majority of participants, 90.12% (1,778 individuals), reported not smoking at all (0 cigarettes per day). Of all participants, 36.6% were between 31 and 35 years old, while only 6% were 20 years or younger. The proportion of people living in an urban area was 67%. Almost 43% of people had an elementary or middle education and 21.4% had an academic education. Only 5.5% of the individuals worked in organizational jobs, while about 56% were housewives. It was found that 15.1% of the respondents suffered from a chronic disease. The results also showed that 23% of the individuals had general studies such as reading novels, magazines, etc. and only 10% of the participants used hookah. In addition, about 83% of hookah users are non-smokers. In other words, people who use hookah are less likely to be smokers (*p* = 0.006). In addition, participants over the age of 30 had the highest average daily consumption at 1.69 (5.66). Women, who made up 63.5% of the sample, had a significantly lower mean (SD) daily consumption at 0.06 (0.50) than men at 2.77 (7.04) (*p* < 0.001). Regarding education, illiterate people had a mean daily consumption of 1.60 (6.94), while participants with higher levels of education had increasingly lower consumption. People working in agriculture or livestock had the highest average daily cigarette consumption 2.84 (6.59), while housewives had the lowest 0.07 (0.77) (*p* < 0.001). The results also showed that participants who did not engage in general study activities had a higher average daily consumption of 1.25 (4.91) compared to 0.39 (5.47) for those who did (*p* = 0.001). On the other hand, there was no significant difference in count of cigarette consumption across BMI (*p* = 0.210), marital status (*p* = 0.119), area of residence (*p* = 0.374), disease history (*p* = 0.640), family history of mental disease (*p* = 0.808), family history of chronic disease (*p* = 0.295) and education categories (*p* = 0.267).

**Table 1:** Number of subjects and mean of cigarette consumption in different cities of Khuzestan, *N* = 1,973.

City	Frequency	Percent of smoker	Light	Moderate	Heavy	Mean ± SD
Abadan	148	9.46%	2.70%	3.38%	3.38%	0.934 ± 3.508
Aghajari	5	20.00%	20.0%	0.0%	0.0%	0.600 ± 1.342
Omidiyeh	30	6.67%	0.0%	0.0%	6.67%	1.667 ± 6.477
Andika	11	0.00%	0.0%	0.0%	0.0%	–
Andimeshk	65	9.23%	6.15%	1.54%	1.54%	0.615 ± 2.805
East Ahwaz	178	8.43%	5.06%	1.12%	2.25%	0.632 ± 2.810
West Ahwaz	176	13.06%	5.11%	2.27%	5.68%	1.523 ± 4.952
Izeh	72	13.89%	5.56%	1.39%	6.94%	1.667 ± 5.523
Baghmalek	55	14.54%	9.09%	3.63%	1.82%	1.091 ± 3.395
Bavi	77	11.69%	3.90%	2.60%	5.19%	1.636 ± 6.048
Behbahan	51	13.73%	13.73%	0.0%	0.0%	0.392 ± 1.133
Hamidiyeh	29	17.24%	10.34%	6.90%	0.0%	0.897 ± 2.320
Khorramshahr	77	9.10%	3.90%	1.30%	3.90%	1.247 ± 5.571
Dezful	235	11.49%	6.38%	2.13%	2.98%	1.264 ± 5.693
Dasht-e-Azadegan	69	7.25%	4.35%	1.45%	1.45%	0.493 ± 2.682
Dehdez	14	21.43%	7.14%	0.0%	14.29%	3.786 ± 9.234
Ramshir	28	14.28%	10.71%	0.0%	3.57%	1.107 ± 3.891
Ramhormoz	55	5.45%	3.64%	0.0%	1.81%	0.346 ± 1.734
Shadegan	99	10.10%	1.01%	4.04%	5.05%	1.400 ± 5.045
Shush	116	6.89%	0.86%	0.86%	5.17%	1.491 ± 6.360
Shushtar	99	6.06%	2.02%	1.01%	3.03%	0.727 ± 3.531
Karun	66	12.13%	6.06%	4.55%	1.52%	0.942 ± 3.085
Gotvand	24	4.17%	0.0%	0.0%	4.17%	1.042 ± 5.103
Lali	8	12.50%	0.0%	0.0%	12.50%	2.500 ± 7.071
Bandar-e Mahshahr	116	6.03%	5.17%	0.0%	0.86%	0.526 ± 3.796
Masjedsoleyman	31	6.46%	3.23%	3.23%	0.0%	0.258 ± 1.125
Haftgel	17	5.88%	5.88%	0.0%	0.0%	0.294 ± 1.213
Hendijan	7	0.00%	0.0%	0.0%	0.0%	–
Hoveizeh	15	13.34%	6.67%	6.67%	0.0%	1.000 ± 2.803
Total	1,973	9.88%	4.71%	1.88%	3.29%	1.050 ± 4.468

SD, Standard deviation.

**Table 2:** Descriptive statistics and comparisons of response components (zero value or count value) by variables.

Variable		Total (*n* = 1,973)			Non-smoker (*n* = 1,778)	Smoker (*n* = 195)	
		*N* (%)	Mean (SD)	*p*-value^¶^		*N* (%)	*p*-value^†^
Age (year)	≤20	121 (6.1%)	0.44 (3.67)	0.002	115 (6.5%)	6 (3.1%)	0.004
	21–25	475 (24.1%)	0.64 (2.87)		437 (24.6%)	38 (19.5%)	
	26–30	654 (33.1%)	0.76 (3.97)		596 (33.5%)	58 (29.7%)	
	>30	723 (36.6%)	1.69 (5.66)		630 (35.4%)	93 (47.7%)	
Sex	Female	1,252 (63.5%)	0.06 (0.50)	<0.001	1,232 (69.3%)	20 (10.3%)	<0.001
	Male	721 (36.5%)	2.77 (7.04)		546 (30.7%)	175 (89.7%)	
BMI	Underweight	110 (5.6%)	1.62 (7.36)	0.210	99 (5.6%)	11 (5.6%)	0.220
	Normal	787 (39.9%)	1.26 (4.84)		696 (39.1%)	91 (46.7%)	
	Overweight	679 (34.4%)	0.90 (3.81)		615 (34.6%)	64 (32.8%)	
	Obese	286 (14.5%)	0.63 (3.20)		266 (15.0%)	20 (10.3%)	
	Extremely obese	111 (5.6%)	1.02 (4.41)		102 (5.7%)	9 (4.6%)	
Marital status	Married	1,419 (71.9%)	1.10 (4.69)	0.119	1,289 (72.5%)	130 (66.7%)	0.085
	Single	554 (28.1%)	0.93 (3.85)		489 (27.5%)	65 (33.3%)	
Education	Illiterate	158 (8.0%)	1.60 (6.94)	0.267	142 (8.0%)	16 (8.2%)	0.346
	Elementary/Middle	848 (43.0%)	1.53 (5.50)		754 (42.4%)	94 (48.2%)	
	Diploma	545 (27.6%)	0.62 (2.85)		503 (28.3%)	42 (21.5%)	
	AD/BSc	397 (20.1%)	0.45 (1.97)		356 (20.0%)	41 (21.0%)	
	MSc/PhD	25 (1.3%)	0.16 (0.55)		23 (1.3%)	2 (1.0%)	
Occupation	Unemployed/student	249 (12.6%)	1.50 (6.20)	<0.001	218 (12.3%)	31 (15.9%)	<0.001
	Organizational	108 (5.5%)	1.81 (5.13)		87 (4.9%)	21 (10.8%)	
	Freelance	420 (21.3%)	2.80 (6.59)		311 (17.5%)	109 (55.9%)	
	Agricultural/livestock	86 (4.4%)	2.84 (8.01)		69 (3.9%)	17 (8.7%)	
	Housewife	1,110 (56.3%)	0.07 (0.77)		1,093 (61.5%)	17 (8.7%)	
Residence area	Rural	654 (33.1%)	1.46 (5.61)	0.374	585 (32.9%)	69 (35.4%)	0.485
	Urban	1,319 (66.9%)	0.85 (3.75)		1,193 (67.1%)	126 (64.6%)	
Disease history	No	1,676 (85.9%)	1.05 (4.48)	0.640	1,508 (84.8%)	168 (86.2%)	0.620
	Yes	297 (15.1%)	1.03 (4.41)		270 (15.2%)	27 (13.8%)	
Family mental disease history	No	1,371 (69.5%)	1.01 (4.16)	0.808	1,234 (69.4%)	137 (70.3%)	0.806
	Yes	602 (30.5%)	1.14 (5.10)		544 (30.6%)	58 (29.7%)	
Family chronic disease history	No	563 (28.5%)	1.43 (5.32)	0.295	502 (28.2%)	61 (31.3%)	0.371
	Yes	1,410 (71.5%)	0.90 (0.90)		1,276 (71.8%)	134 (68.7%)	
General study	No	1,519 (77.0%)	1.25 (4.91)	0.001	1,351 (76.0%)	168 (86.2%)	0.001
	Yes	454 (23.0%)	0.39 (5.47)		427 (24.0%)	27 (13.8%)	
Hookah consumption	No	1,838 (89.4%)	1.02 (4.41)	0.006	1,666 (93.7%)	172 (88.2%)	0.004
	Yes	135 (10.6%)	1.46 (5.23)		112 (6.3%)	23 (11.8%)	

SD, Standard deviation.

¶ *p-value conducted from Chi-square test.*

† *p-value conducted from Kruskal-Wallis/Mann–Whitney-U test.*

Regardless of the adjusted models for the covariate effect, the abundance of zeros in Figure 1 suggests that the use of ZI models can be appropriate for these data. When comparing the six two-level models using the indices, it was also found that the ZI models performed better than the simple count models in terms of model fit. In addition, of the three models with zeros, the ZICMP and ZINB models had better indices than the ZIP model. A further comparison between the ZICMP and ZINB models showed only minor differences in their indices, with the ZICMP model being superior in all aspects. All goodness-of-fit indices for the six models are listed in Table 3. Therefore, the results of the two-level ZICMP model are used for the interpretation of the results in this study.

**Table 3:** Comparison of models fit, *n* = 1,973.

Fit statistics	Model					
	TL-P	TL-NB	TL-CMP	TL-ZIP	TL-ZINB	TL-ZICMP
P	25	26	26	50	51	51
LL	−3,486.847	−1,167.437	−1,665.115	−1,298.044	−1,083.787	−1,081.795
D	6,973.694	2,334.874	3,330.23	2,596.088	2,167.574	2,163.59
AIC	7,023.694	2,386.874	3,382.230	2,696.088	2,269.574	2,265.590
BIC	7,163.377	2,532.144	3,527.500	2,746.254	2,554.527	2,550.491
MSE	15.199	21.859	16.882	16.643	16.338	16.331
Dispersion parameter: *r* or *ʋ*	–	0.071	0.04	–	1.75	0.38

P, number of parameters in the fitted model; LL, Log-Likelihood; D, Deviance (D = Residual Deviance − Null Deviance); AIC, Akaike information criterion (AIC = −2 × LL + 2 × *p*); BIC, Bayesian information criterion (BIC = −2 × LL + *p* × ln (*n*)); MSE, Mean square error $\left(MSE=\frac{1}{N}\overset{N}{\underset{i=1}{\sum }}{\left(y_{i}-\hat{y_{i}}\right)}^{2}\right)$ .

The results of the TL-ZICMP model for the count part show that, age, gender, body mass index (BMI), and education are significantly associated with daily smoking (Table 4). The fitted model estimates that male smokers are expected to consume exp. (1.175) = 3.24 times more cigarettes a day compared to female smokers. Obese smokers have a nearly 48% lower rate of daily smoking than underweight individuals (exp (−0.663)). Also, there is an inverse relationship between education level and daily smoking consumption; higher education levels correspond to lower daily cigarette consumption. Specifically, individuals with MSc or PhD degree are 91.74% less likely to smoke daily compared to illiterate individuals (RR = 0.083). Additionally, daily cigarette consumption increases with age, with individuals over 30 consuming twice as many cigarettes per day as those under 20 (RR = 2.155), although this difference is not statistically significant at the 0.05 level.

**Table 4:** Parameter estimates and standard errors for TL-ZICMP regression model.

		TL-ZICMP			
Variable		Zero-Inflation part	Count part	OR	RR
		Est. (SE)^Sig. level^	Est. (SE)^Sig. level^		
Age (year)	≤20	Ref.	Ref.	1.0000	1.0000
	21–25	−0.44109 (0.50504)	0.12075 (0.43410)	0.6433	1.1283
	26–30	−0.59867 (0.51041)	0.56700 (0.44347)	0.5495	1.7630
	>30	−1.01933 (0.51929)^*^	0.76794 (0.44167)^1^	0.3608	2.1553
Sex	Female	Ref.	Ref.	1.0000	1.0000
	Male	−2.26432 (0.56626)^***^	1.17575 (0.49412)^*^	0.1039	3.2406
BMI	Underweight	Ref.	Ref.	1.0000	1.0000
	Normal	−0.22026 (0.38289)	−0.28213 (0.26514)	0.8024	0.7542
	Overweight	0.09111 (0.39402)	−0.25832 (0.27551)	1.0954	0.7723
	Obese	0.20643 (0.44966)	−0.66341 (0.32452)^*^	1.2293	0.5151
	Extremely obese	0.08046 (0.54884)	−0.08630 (0.37434)	1.0838	0.9173
Marital status	Married	Ref.	Ref.	1.0000	1.0000
	Single	−0.36787 (0.22366)	0.12694 (0.16951)	0.6922	1.1353
Education	Illiterate	Ref.	Ref.	1.0000	1.0000
	Elementary/Middle	0.35701 (0.34184)	−0.24852 (0.23027)	1.4291	0.7800
	Diploma	1.08590 (0.38194)^**^	−0.65698 (0.27284)^*^	2.9621	0.5184
	AD/BSc	0.59431 (0.40913)	−1.12731 (0.29517)^***^	1.8118	0.3239
	MSc/PhD	0.05623 (1.20299)	−2.49417 (0.92141)^**^	1.0578	0.0826
Occupation	Unemployed/student	Ref.	Ref.	1.0000	1.0000
	Organizational	−0.58931 (0.37386)	−0.29927 (0.28125)	0.5547	0.7414
	Freelance	−0.39806 (0.26251)	−0.27259 (0.19532)	0.6716	0.7614
	Agricultural/livestock	0.11483 (0.37260)	−0.24061 (0.26440)	1.1217	0.7861
	Housewife	0.55142 (0.62593)	0.01011 (0.50452)	1.7357	1.0102
Residence area	Rural	Ref.	Ref.	1.0000	1.0000
	Urban	−0.22949 (0.19590)	−0.21474 (0.14851)	0.7949	0.8068
Disease history	No	Ref.	Ref.	1.0000	1.0000
	Yes	0.28857 (0.24677)	0.29239 (0.19449)	1.3345	1.3396
Family mental disease history	No	Ref.	Ref.	1.0000	1.0000
	Yes	−0.10962 (0.19167)	0.03155 (0.14003)	0.8962	1.0321
Family chronic disease history	No	Ref.	Ref.	1.0000	1.0000
	Yes	−0.26693 (0.18977)	−0.11941 (0.14287)	0.7657	0.8874
General study	No	Ref.	Ref.	1.0000	1.0000
	Yes	0.51302 (0.27069)^1^	−0.30323 (0.22387)	1.6703	0.7384
Hookah consumption	No	Ref.	Ref.	1.0000	1.0000
	Yes	0.18491 (0.27152)	−0.14772 (0.21535)	1.2031	0.8627
Variance (SD)		3.365e−10 (1.834e−05)	1.658e−08 (0.0001288)	–	–

Results reported as parameter estimation (Standard Error).

Ref., Reference level.

TL-ZICMP, Two-Level Zero-Inflated Conway–Maxwell Poisson.

Significant codes: (‘***’ < 0.001); (‘**’ < 0.01); (‘*’ < 0.05); (‘1’ < 0.1).

In the zero-inflated part of the model, age, gender, and education also influence the likelihood of being a non-smoker. Older individuals (>30 years) are almost 64% less than those aged 20 or younger who do not smoke (OR = 0.361). Females are more likely to be non-smokers, with a significantly higher probability of not smoking at all (1/exp. (−2.26) = 9.56). Education also plays a crucial role; individuals with a diploma are nearly three times more likely to be non-smokers than illiterate individuals (OR = 2.962). There was no significant association between daily cigarette consumption and disease history in both parts of the model.

## 4 Discussion

In this study, the data on daily cigarette consumption among young people aged 18 to 35 years in Khuzestan province, Iran, were modeled using count models and count models with excess zeros. The results were evaluated in terms of the factors affecting smoking behavior, and an appropriate model was created to analyze this process. To explain the underlying heterogeneity of the population, we used a two-level random-effects count model.

Given the high prevalence of zeros (indicating non-smoking individuals), the model comparison showed that models with excess zeros are appropriate for assessing outcomes. Furthermore, the comparison of models with excess zeros in a two-level framework showed that the ZINB and ZICMP models provided satisfactory results compared to the ZIP model. The comparison between the ZINB and ZICMP models revealed no significant differences. Previous studies have supported the use of the ZINB model over the ZIP model due to the presence of over-dispersion (43). For instance, a 2020 study found that the NB model performed better than the P model when fitting adolescent smoking data (44). Fatih Tuzen also demonstrated that models such as ZINB and Negative Binomial Hurdle (NBH) were superior to NB, P, and Poisson Hurdle (PH) models in analyzing young people’s daily cigarette consumption (34). Past studies have used well-known count models for the determinant cigarettes or tobacco, but none have considered the CMP model. Furthermore, this model does not converge for some data sets (42). In our study, however, it was fitted to the data despite the large number of observations and model variables. The indices of ZICMP model was very similar to ZINB model, however, the ZICMP model performed slightly better on all indices. Young et al. (35) compared different ZI count models of health insurance coverage in Hawaii using the AIC and BIC indices. Their results suggest that of the four models—ZIP, ZINB, ZICMP and Zero-inflated Generalized Poisson (ZIGP)—the ZINB model provides the best fit to the data (35).

Additionally, Ghorbani et al. showed that the ZICMP model for DMFT data in a three-level structure was better than the ZINB model, which aligns with our findings (45). In another study from 2022, the three count model P, NB and CMP were compared using schizophrenia data and it was found that the NB model fitted best based on the main effects model (46). A 2023 study comparing ZINB and Zero-Inflated Discrete Weibull (ZIDW) models found that ZIDW had a better fit (20). Given the acceptable performance of ZINB and ZICMP in our study and the ability of the ZICMP model to account for both over- and under dispersion, it is reasonable to assume that the ZICMP model can be used effectively in contexts where the ZINB model performs well (47).

From the perspective of data description, our study revealed that the prevalence of current smoking among 18–35-year-olds in Khuzestan is 1.60% for females and 24.27% for males. These figures are slightly higher than those of the 2016 national STEPs survey, which showed a prevalence of 1.10 and 24.10% for ever smoked cigarettes and 0.30% and 17.8 for current cigarette smoking for women and men, respectively (10). In addition, the prevalence of smoking in the 18–24 and 25–34 age groups in Iran was 2.3 and 7.2%, respectively. In our study, higher percentages of about 7.5 and 11% for 18–25 and 26–35 age groups, respectively, were found for Khuzestan (10). Studies consistently show that men tend to consume more cigarettes per day than women, which is supported by our findings (3, 48, 49). According to Parami et al., the rate ratio (RR) of daily cigarette consumption was nine times higher in male students than in female students (44). Similarly, Moghimbeigi et al. found that the RR in 15–20 year old Iranian adolescents was almost four times higher in males than in females (6). However, a study in seven European countries found that 15-year-old girls are slightly more likely to smoke daily than boys (50).

Several studies have investigated the correlation between education level and smoking habits (51). Our study identified education as a significant variable for daily cigarette consumption, with smoking decreasing as education levels increased. This finding is consistent with other studies (20, 24). A 2020 study in Japan reported that the prevalence of smoking was lowest among college graduates compared to participants with less education, with the highest prevalence among those with a junior high school degree (52). Garrett et al. also found that the prevalence of smoking decreases with increasing educational levels among men and women aged 25–44 years (53). However, some studies have found no correlation between educational level and smoking (34). It is worth noting that many studies on smoking focus on specific age groups, which can lead to a more homogeneous distribution of participants’ educational levels. In contrast, our study, which includes participants aged 18–35, captures a broader age range and thus greater academic diversity. This diversity is important to understand how the prevalence of smoking and age of smoking initiation vary between educational levels.

Age was another significant covariate in our study. Some studies have examined the relationship between the age at which smoking was started and smoking intensity in adulthood (54). These studies have shown that people who started smoking at a younger age tend to have higher smoking rates than those who started at an older age, with high cigarette consumption associated with adverse health outcomes, including death (55). Our results indicate that with increasing age, the probability of being a non-smoker decreases, while cigarette consumption among smokers increases. Our results also showed that around 5% of people aged 20 and under are smokers. A study in the United States found that the proportion of people who started smoking at age 22 and 23 increased (about 22%) in 2018 compared to 2002 (56). However, a study of the 15–20 age group showed that the probability of being a smoker decreases with increasing age (6).

On the other hand, the results of our study are consistent with previous research on certain covariates, including occupation and hookah use, although these variables were not statistically significant. Previous studies indicate that many young people have a positive attitude toward hookah smoking, believing it to be less harmful, non-addictive, and socially acceptable (57, 58). Our study found that nearly 83% of hookah smokers are non-smokers. Additionally, work-related stress is a factor that can lead to smoking initiation (59). A study of smoking among young people (aged 15–24) in Turkey found that employed young people were about three times greater likely to be smokers than unemployed young people, and this figure was almost twice as high in our study (34). Conversely, Moghimbeigi et al. found that the probability of becoming a smoker is higher among unemployed individuals and housewives in the 15–20 age group compared to students (6). Ultimately, it can be said that the results of the various studies on smoking can vary somewhat depending on the target society. Also, our study indicated that the smoking risk among individuals aged 18–35 is slightly higher than in previous studies. This suggests that, despite policies to reduce smoking and promote cessation, the number of smokers remains significant, highlighting the need for more effective interventions.

### 4.1 Limitations and recommendations

Future studies should include additional factors such as the social status of the family, tobacco consumption within the family and the economic status of the family. In this study, more than 40% of the data on family income was missing, so it had to be excluded from the analysis. Future studies should examine income as a socioeconomic status variable alongside other important factors. Understanding the age at which smoking is started could provide valuable insights for policy makers. This study focused on people aged 18–35 years. By including a broader age range, study can provide more comprehensive insights into smoking patterns across different age groups. Given the growing concern about drug use, it is suggested that similar studies be conducted on other drugs, particularly cannabis/hashish and opium. In addition, it would be valuable to investigate effective strategies for deterring young people and the potential of a ‘truth’ style anti-tobacco campaign and to evaluate government initiatives to raise awareness of these issues.

## 5 Conclusion

The use of advanced count models capable of handling numerous zeros and overdispersion is crucial for accurately analyzing trends in cigarette consumption across different population groups. The results indicate that factors such as older age, lower education levels, and gender differences influence smoking behavior. As early initiation of smoking can lead to long-term dependence and health problems, prevention strategies that delay the onset of smoking, especially among men, and promoting education among adolescents can be effective in reducing the risk and consumption of cigarettes. Educational campaigns that raise awareness of the risks associated with smoking may also help to reduce consumption in different population groups. However, further research should consider additional socioeconomic variables and include a broader age range to better understand smoking behavior.

## Data Availability

The raw data supporting the conclusions of this article will be made available by the authors without undue reservation.
